# Impact of teacher teaching quality evaluation on the learning motivation of pre-service English teachers: a chain mediation analysis of school climate perception and academic anxiety

**DOI:** 10.3389/fpsyg.2026.1889668

**Published:** 2026-08-03

**Authors:** Yiyin Li

**Affiliations:** School of Education, City University of Macau, Macao, Macao SAR, China

**Keywords:** teaching quality evaluation, learning motivation, school climate perception, academic anxiety, chain mediation, pre-service English teachers

## Abstract

**Introduction:**

This study examined the associations among pre-service English teachers' evaluations of teacher teaching quality, perceived school climate, academic anxiety, and learning motivation.

**Methods:**

Guided by social cognitive theory and sociocultural theory, we tested an association-based chain mediation model using cross-sectional self-report questionnaire data from 1,643 pre-service English teachers in China.

**Results:**

More positive evaluations of teaching quality were associated with higher learning motivation and more positive school climate perceptions, and with lower academic anxiety. School climate perception and academic anxiety showed significant indirect associations between teaching quality evaluation and learning motivation, including a small but statistically significant sequential indirect pathway.

**Discussion:**

Pre-service English teachers who evaluated teaching quality more positively also tended to perceive a more supportive school climate, report less academic anxiety, and demonstrate stronger learning motivation. Given the cross-sectional self-report design, these findings represent statistical associations rather than causal or temporal effects. The results underscore the relevance of teaching quality, supportive learning environments, and academic-emotional support in teacher education.

## 1 Introduction

Research in educational psychology has increasingly examined how students' evaluations of teaching, perceptions of school climate, academic emotions, and motivation are related within higher education. These variables are particularly important in teacher education because pre-service teachers are not only students in the present but also future classroom practitioners. Their evaluations of teaching quality may shape how they interpret effective pedagogy, how they experience the learning environment, and how strongly they remain motivated to develop professional competence.

Teacher teaching quality evaluation, as a core component of education quality monitoring, was first proposed by Professor HW Marsh in 1986 with the “Student's Evaluations of Educational Quality” (SEEQ). This scale comprehensively evaluates teachers' teaching performance from the student's perspective and covers nine dimensions (Marsh, 1986). Since its inception, this scale has been widely used and validated globally (Marsh et al., 1997). For example, in Hong Kong, SEEQ was successfully introduced and its universality in different cultural contexts was demonstrated (Hau and Marsh, 2004). In China, domestic scholars have adapted and expanded SEEQ to suit the needs of different educational levels (Ju, 2001; Zou and Chen, 2013). Related studies have shown that students' evaluation of teachers' teaching quality not only affects teachers' teaching strategies, but is also closely related to students' learning motivation and academic achievement (Feng, 2017; Liu et al., 2019; Zhang et al., 2023).

Meanwhile, school atmosphere perception, as an important factor influencing students' learning experience, has received increasing attention in recent years. Research on school atmosphere covers multiple dimensions such as physical environment, teacher-student relationship, peer relationship, and school culture. These factors together shape students' overall learning experience and psychological development (Li et al., 2020). International research shows that a positive school atmosphere can effectively improve students' academic performance and have a positive impact on their mental health (Wang and Eccles, 2013; Fletcher et al., 2008). In China, researchers have also explored the relationship between school atmosphere and students' learning engagement and revealed the differential impact of school atmosphere on the development of different student groups. For example, a good school atmosphere has a significant protective effect on disadvantaged student groups (Thapa et al., 2013; Liu, 2017; Hou et al., 2018). In addition, school atmosphere perception is also considered an important factor affecting college students' mental health, academic performance and social behavior (Way and Robinson, 2003; Cao and Liu, 2018). However, research on future teachers—teacher trainees—is relatively scarce, especially how their perception of school atmosphere affects their learning motivation and future teaching practice, which is still a topic worthy of further exploration.

Academic anxiety, as an important component of students' academic emotions, has attracted widespread research interest in recent years. Academic anxiety, including test-related anxiety, can affect students' motivation and cognitive resources (Pekrun, 2006; Gao and Liu, 2021). Academic emotions are closely associated with students' learning engagement and academic achievement (Wang and Wu, 2023; Hu et al., 2022). Although research on academic anxiety is relatively abundant, more attention still needs to be paid to academic anxiety among teacher trainees and its mechanisms of action on learning motivation.

Finally, learning motivation is a crucial intrinsic driving force behind students' learning behavior and has become a research hotspot in educational psychology and pedagogy in recent years. The theoretical development of learning motivation encompasses multiple stages, from reinforcement theory to achievement goal theory, and has been gradually applied to educational practice (Ryan and Deci, 2000; Dweck and Leggett, 1988; Skinner, 1953). Multiple classifications of learning motivation, such as intrinsic and extrinsic motivation, biological and social motivation, reveal its diversity and complexity in the learning process (Li and Yan, 1993; Zhao, 2004). However, despite a wealth of theoretical and empirical research on learning motivation, studies on the learning motivation of teacher trainees, particularly its relationship with teacher evaluation of teaching quality, perceived school atmosphere, and academic anxiety, remain insufficient. Existing literature shows that learning motivation plays a central role in students' learning process, and a deep understanding of its interaction with key variables in educational practice is of great significance for promoting students' all-round development and improving the quality of education (Amabile et al., 1994).

This study therefore examines the relationships among pre-service English teachers' subjective evaluations of teacher teaching quality, perceived school climate, academic anxiety, and learning motivation. Specifically, it tests whether school climate perception and academic anxiety statistically mediate the association between teaching quality evaluation and learning motivation. Given the cross-sectional design, the model is treated as an association-based mediation model rather than proof of causal ordering.

## 2 Literature review

Recent research has also linked teacher motivation with the quality of education at the secondary and higher-secondary levels (Anghel, 2024). Previous studies have linked teaching quality, teaching style, and learning environment with student engagement and motivation. However, this literature must be interpreted carefully because students' evaluations of teaching quality are perception-based measures rather than objective observations of instruction. Gonţa and Tripon (2021), for example, showed that students evaluate teaching practices in relation to their expectations and learning experiences. In the present study, teacher teaching quality evaluation is therefore operationalized as pre-service English teachers' subjective evaluation of course teaching quality using SEEQ-based items, not as an externally observed measure of teaching performance. Recent studies have also highlighted the importance of feedback processes in educational contexts (Tünkler, 2021).

Anghel (2017) further tries to give the broader understanding of the relevance of modern teaching assessment and particularly with respect to learning motivation. In this particular study, the influence of different ways of teaching and the capability of the teacher to adjust to various situations and ultimately influence the students to be motivated to learn, will be discussed. The research findings hint at the idea that the evaluation instruments applied to the learning process within the educational environment should not solely refer to the academic achievement but to the motivational factors which help to shape the learning experience in its entirety. Likewise, the role of teaching styles, especially in rural and remote schools, is of significant priority to Salipong-Ubayubay (2025). The paper associates certain motivational practices employed by teachers in achieving better results in learning, which would ascertain the notion that different types of teaching style can be essential in motivating students.

Wiangga and Febriyanto (2024) goes further to examine the impact of the teaching styles on the motivation of learners in secondary schools, with reference to the English language learning. As pointed out in the current paper, interactive and student-centered instructional methods have a positive impact on students, and are more likely to encourage learners than the conventional, lecture-based methods of teaching. The results by Wiangga-Febriyanto are reminiscent of the works by Popova et al. (2016) who opine that teacher development especially in motivation should be incorporated to boost teaching effectiveness.

Lunkina et al. (2023) discuss how the quality of the teaching process is one of the predictors of student engagement, wellbeing, and performance and offer empirical data to back the same scenario: high-quality teaching is correlated with better performance of students. In their study, they highlight how the quality of education, when it is guided by student needs and interests, can not only promote student engagement but also academic performance. This is essential since it highlights the importance of evaluating the situational conditions that determine the learning process which is normally neglected in the conventional evaluation.

Rothinam et al. (2024) give a thorough overview of the literature about the factors which affect teacher motivation and list both intrinsic and extrinsic factors as the motivators. According to their findings, intrinsic determinants, like personal satisfaction and enthusiasm in teaching, are more considerable as opposed to extrinsic rewards in the process of teachers remaining motivated in the long run. But, they also admit that the external rewards (salary and career promotion) also may be added to intrinsic motivation and allow to achieve better teacher performance.

It is a critical study of teacher and administrator assessments of the law on teaching careers by Sahin (2023), which also dwells on the law as a determinant of the teaching practices and its effects on motivation of teachers. The research criticizes the current evaluation systems on the basis of failing to focus on psychological and motivation facets of teaching and as such, these systems are not able to reflect the realities of teacher performance and teacher motivation.

The article by Pakino and Ubayubay (2024) explores the concept of job satisfaction and motivation in teachers working in the alternative learning system, namely, marginalized communities. This paper indicates that the motivation of the teachers in alternative learning environments contrasts very well with that of a traditional school setting based on personal gratification of teaching, as well as the demands of education in non-traditional schools. This brings another twist into the current discussion of the ways in which teacher motivation may differ in educational settings.

Yahya et al. (2023) examine the relationship between the quality of teaching and school facilities that provide helpful information regarding the impacts of the external factors, including the learning environment, on teacher motivation and student performance. The research highlights the relevance of the favorable physical and psychological climate in strengthening the motivation of the teacher or student learning. Likewise, Boyle and Cook (2023) make their contributions to the debate on teaching quality and pay attention to the developmental assessment of teaching practices. They underline in their research that teacher motivation and enhancement of teaching results are achieved by continuous professional growth and feedback created by an individual teacher.

Vučinić et al. (2023) analyze the motivational behaviors of mathematics teachers through the lens of the students, indicating that interaction between teachers and students is a key consideration when motivating students. The work gives importance to the influence of teacher enthusiasm and encouragement in a positive learning environment, which also justifies that teacher behavior plays a critical role in determining the motivation of students.

Buribayev et al. (2025) interpret the establishment of the research culture in the future elementary school teachers. The paper focuses on the idea of motivating the future teachers by exposing them to research and thinking at a very young age, which will enhance their teaching methods and general satisfaction with the job. This contributes to the use of the literature as the teacher motivation efforts are connected to the professional growth and research activity.

Lastly, Imam-Wiradendi (2025) explores how the teaching style of a teacher affects the learning motivation of students in the SMAN 1 Maja. Findings indicate that instructional strategies; those using student-centered teaching methods, e.g., cooperative learning, and inquiry-based instructions are better in encouraging students than the traditional teaching instruction. This supports the study by Wiangga and Febriyanto (2024), and also proves the significance of the teaching style in student motivation.

### 2.1 Theoretical framework and conceptual model

The theoretical framework integrates Bandura's (1986) social cognitive theory with Vygotsky's (1978) sociocultural theory. Social cognitive theory explains motivation through reciprocal interactions among personal beliefs, behavior, and environment. In this context, the positive feedback that students may give on the teaching quality could be linked with increased motivational beliefs, as high quality teaching would provide clearer goals, teaching support, and effective modeling of teaching and learning practices. The view of learning motivation is complemented by the Socio-cultural theory which focuses on the social interaction and institutional context of learning motivation. As such, a supportive school climate can be used as a contextual factor to connect teaching quality assessment with motivation.

According to Bandura's model, perceived quality of teaching can be related to students' self-regulation and self-efficacy processes. The pre-service teachers' interpretation of learning tasks as manageable and worthwhile tasks could be facilitated by clear organization, constructive interaction, teacher's enthusiasm and fair assessment. The perceptions may also be linked to a reduction in academic anxiety as those who felt they had been guided more clearly and had more predictable academic expectations are less likely to think of academic demands as threatening. It sets the theoretical foundation to expect negative relationships between the teaching quality evaluation and academic anxiety, and positive relationships between the academic anxiety and learning motivation.

The school climate is one of the significant social contexts for learning, according to Vygotsky's sociocultural theory of learning. A pre-service teacher's sense of belonging, active participation, and seeking out assistance from a peer group, school support, and resource availability may foster a sense of belonging and support for a pre-service teacher. In this model, the school climate perception is placed in the middle due to the possibility that the evaluation of teaching quality may be linked to the school climate perception. Academic anxiety is included as a second mediator as it is possible that a positive climate might be related to less anxiety, which then might relate to increased motivation. The outcome is a theoretically based mediation model, not a causal process supported by temporal evidence.

### 2.2 Research hypothesis

Based on the above theoretical analysis and a review of previous literature, this study proposes the following hypotheses:

H1: Teacher evaluations of teaching quality are positively associated with school climate perception.
H2: Teacher evaluation of teaching quality is negatively associated with academic anxiety.
H3: School climate perception is negatively associated with academic anxiety.
H4: School climate perception is positively associated with learning motivation.
H5: Academic anxiety is negatively associated with learning motivation.
H6: Teacher evaluation of teaching quality is positively associated with learning motivation.
H7: School climate perception statistically mediates the association between teacher evaluation of teaching quality and learning motivation.
H8: Academic anxiety statistically mediates the association between teacher evaluation of teaching quality and learning motivation.
H9: School climate perception and academic anxiety form a statistically significant chain mediation pathway between teacher evaluation of teaching quality and learning motivation.

## 3 Methodology

### 3.1 Characteristic analysis of demographic samples

A total of 1,789 questionnaires were returned. After excluding responses with excessively short completion times and identical answer patterns, 146 cases were removed, leaving 1,643 valid responses for analysis. The effective response rate was 91.8%. The characteristics of the valid sample are shown in Table 1.

**Table 1:** Characteristic analysis of the demographic sample.

Variable	Options	Frequency	Percent
Gender	Male	537	32.7
	Female	1,106	67.3
Is the child an only child?	Yes	1,040	63.3
	No	603	36.7
Do you have internship, part-time, or work experience?	Yes	1,239	75.4
	No	404	24.6
Have you received any scholarships or other academic awards?	Yes	445	27.1
	No	1,198	72.9
School level	National 985/211	337	20.5
	Non-985/211 first-tier universities	924	56.2
	Second-tier universities that are not 985/211	382	23.3
Overall satisfaction with your school	Very satisfied	915	55.7
	Quite satisfied	345	21
	Generally	222	13.5
	Not very satisfied	105	6.4
	Very dissatisfied	56	3.4
Grade and class	Freshman	397	24.2
	Sophomore	451	27.4
	Junior	398	24.2
	Senior year	299	18.2
	Graduate	98	6
Father's highest education	To receive formal education	67	4.1
	Primary school or below	223	13.6
	Junior high school or equivalent education	258	15.7
	High school or equivalent education	282	17.2
	Vocational school or technical school	389	23.7
	Associate's degree (or equivalent)	215	13.1
	Undergraduate (Bachelor's Degree)	180	11
	Master's degree (Master's degree)	13	0.8
	Doctoral student (doctoral degree)	16	1
Mother's highest education	To receive formal education	95	5.8
	Primary school or below	76	4.6
	Junior high school or equivalent education	202	12.3
	High school or equivalent education	266	16.2
	Vocational school or technical school	387	23.6
	Associate's degree (or equivalent)	369	22.5
	Undergraduate (Bachelor's Degree)	197	12
	Master's degree (Master's degree)	33	2
	Doctoral student (doctoral degree)	18	1.1
Family (parents) total monthly income range	Less than 5,000 yuan	299	18.2
	5,000 to 15,000 yuan	854	52
	15,000 to 30,000 yuan	310	18.9
	30,000 to 50,000 yuan	66	4
	More than 50,000 yuan	114	6.9
The most important ability or quality to become an excellent English teacher	A. Profound knowledge of the English language	210	8.6
	B. Excellent communication skills	542	22.1
	C. High levels of patience and empathy	325	13.3
	D. Innovative teaching methods	351	14.3
	E. Good classroom management skills	312	12.7
	F. Willingness to continue learning and self-improvement	384	15.7
	G. Cultural sensitivity and diversity awareness	327	13.3

Table 1 presents the demographic characteristics of the sample. Regarding gender, there are 537 males (32.7%) and 1106 females (67.3%), indicating a relatively larger number of female respondents. Regarding whether they are only children, 1040 are only children (63.3%), and 603 are not only children (36.7%), indicating that the majority of respondents are only children. Regarding internship, part-time, or work experience, 1239 have such experience (75.4%), and 404 have not (24.6%), indicating that most respondents have such experience. Regarding whether they have received scholarships or other academic awards, 445 have received such awards (27.1%), and 1198 have not (72.9%), indicating that a relatively larger number of respondents have not received such awards. Regarding the level of the schools, the majority (56.2%) are non-985/211 first-tier universities. Regarding overall satisfaction with their schools, a relatively large number (55.7%) are very satisfied. Regarding their grade and class, first-year, second-year, and third-year students make up the majority (nearly 80%). As for the father's highest education level, it mainly ranges from primary school or below to a bachelor's degree. Regarding the mother's highest education level, it mainly ranges from junior high school or equivalent to a bachelor's degree. Regarding the total monthly income of the family (parents), 5,000 to 15,000 yuan is the main income level, accounting for 52%. Regarding the most important abilities or qualities for becoming an excellent English teacher, excellent communication skills and a willingness to continuously learn and improve oneself are relatively important.

### 3.2 Research tools and reliability testing

Since the measurement tools used in this study were all scales with many items, SPSS 25.0 software was used to perform statistical analysis on the Cronbach's α coefficients of the four scales to ensure internal consistency and reliability of the data. By calculating the Cronbach's α coefficient for each scale, we were able to determine whether the measurement results of each variable met the ideal reliability standard. The specific results are presented in Table 2.

**Table 2:** Reliability test.

Scale	Dimension	N of items	Cronbach's alpha	
Teacher teaching quality evaluation	Learning value	4	0.868	0.957
	Teachers' enthusiasm	4	0.891	
	Clear organization	4	0.868	
	Group interaction	4	0.895	
	Teacher–student relationship	4	0.808	
	Coverage range	4	0.893	
	Exam scoring	3	0.872	
	Essay reading	2	0.804	
	Course difficulty	3	0.834	
	Overall evaluation	2	0.844	
School atmosphere perception	Classmate relationship	10	0.957	0.965
	Innovation	4	0.881	
	Participate in decision-making	7	0.888	
	Resource sufficiency	10	0.961	
	Student support	7	0.946	
Academic anxiety	Classroom-related emotions	8	0.928	0.966
	Learning related emotions	8	0.897	
	Exam-related emotions	8	0.947	
	Emotions related to academic writing	8	0.942	
Learning motivation	Challenging	11	0.965	0.969
	Passion	3	0.885	
	Relying on others' evaluation	7	0.947	
	Select a simple task	5	0.942	
	Focus on interpersonal competition	2	0.859	
	Seeking returns	2	0.864	

This study used Marshel's 1997 SEEQ questionnaire, which includes nine dimensions: learning value, teacher enthusiasm, organizational clarity, group interaction, teacher-student relationship, breadth of coverage, test scores, essay and reading comprehension, course difficulty, and overall evaluation, totaling 34 items. Each item was rated using a 5-point Likert scale, ranging from “strongly disagree” to “strongly agree.” High scores indicate positive evaluations of teacher teaching quality. The scale's Cronbach's α coefficient in this study was 0.957, indicating high reliability. The Cronbach's alpha coefficients for the dimensions of learning value, teacher enthusiasm, clear organization, group interaction, teacher-student relationship, breadth of coverage, exam scores, essay reading, course difficulty, and overall evaluation were 0.868, 0.891, 0.868, 0.895, 0.808, 0.893, 0.872, 0.804, 0.834, and 0.844, respectively, all greater than 0.7, indicating that the reliability of these dimensions is good.

This study used the School-Level Environment Questionnaire (SLEQ) developed by Johnson and Stevens (2006). Because the original SLEQ was designed to measure teachers' perceptions of their professional work environment, the wording was adapted for pre-service English teachers. For example, terms such as “teacher” and “colleague” were replaced with “classmate” where the item referred to peer relations, and “principal” was replaced with “mentor” where appropriate. These modifications mean that the instrument measures students' perceived school climate in a teacher-education learning context rather than teachers' organizational climate. To address the validity concern created by this adaptation, the revised scale was examined through reliability analysis, exploratory factor analysis, and confirmatory factor analysis. The adapted scale showed strong internal consistency (Cronbach's α = 0.965), clear five-factor structure, acceptable model fit, convergent validity, and discriminant validity, supporting its use in this sample while recognizing that further validation in other contexts is still needed.

This study used the Achievement Emotions Questionnaire (AEQ) developed by Pekrun et al. (2011). The original questionnaire includes classroom-related emotions, learning-related emotions, and exam-related emotions. Considering that thesis writing is an important academic requirement and anxiety source for undergraduate teacher trainees, eight thesis-writing-related items were added with reference to studies on undergraduate writing anxiety (Wang, 2018; Ning et al., 2022, 2023; Li and Xu, 2012). The added items focused on worry, tension, and perceived pressure during thesis preparation and writing. The expanded academic anxiety scale therefore contained four dimensions and 32 items. Reliability, exploratory factor analysis, and confirmatory factor analysis were used to evaluate whether the modified structure was acceptable. The overall Cronbach's α coefficient was 0.966, and all four dimensions showed acceptable reliability and validity indicators.

This study measured learning motivation using a revised learning motivation scale, referencing the work of Chi and Xin (2006) and Amabile et al. (1994). The scale comprises six dimensions: challenge, enthusiasm, reliance on peer evaluation, choosing easy tasks, focus on interpersonal competition, and pursuit of reward, with a total of 30 items. Each item was scored using a 5-point Likert scale, with higher scores indicating higher levels of learning motivation. The scale's Cronbach's α coefficient in this study was 0.969, indicating extremely high reliability. The Cronbach's α coefficients for the dimensions of challenge, reliance on peer evaluation, and choosing easy tasks were 0.965, 0.947, and 0.942, respectively, all greater than 0.9, indicating good reliability for these dimensions. The Cronbach's α coefficients for the dimensions of enthusiasm, focus on interpersonal competition, and pursuit of reward were 0.885, 0.859, and 0.864, respectively, all greater than 0.7, indicating good reliability for these dimensions.

### 3.3 Data processing

The questionnaire was administered online through Wenjuanxing after standardized instructions had been provided. Participation was voluntary and anonymous, and the survey introduction explained the academic purpose of the study. The data were screened for invalid responses before analysis. IBM SPSS Statistics, version 25.0 (IBM Corp., Armonk, NY, United States) was used for descriptive statistics, reliability testing, exploratory factor analysis, correlation analysis, and Harman's single-factor test. IBM SPSS Amos, version 24.0 (IBM Corp., Armonk, NY, United States) was used for confirmatory factor analysis, structural equation modeling, and bootstrap testing of indirect effects.

To strengthen the interpretation of the adapted measures, reliability analysis, exploratory factor analysis, confirmatory factor analysis, convergent validity, and discriminant validity were reported for each scale. These analyses were used not only to support internal consistency but also to examine whether the modified instruments retained interpretable factor structures in the pre-service English teacher sample.

## 4 Results

### 4.1 Exploratory factor analysis

Exploratory factor analysis is primarily used to measure the construct validity of a scale. Construct validity refers to the consistency between the experiment and the theory, that is, whether the experiment truly measures the hypothesized (constructed) theory. If the correspondence between factors and measurement items matches expectations, it indicates construct validity. When using factor analysis for validity analysis, it is necessary to first determine whether the conditions for factor analysis are met. Generally, two conditions need to be met: first, the KMO value needs to be greater than 0.7; second, the significance of Bartlett's test of sphericity needs to be less than 0.05. If these two conditions are met, it indicates a strong correlation between the observed variables, making it suitable for factor analysis.

Table 1 shows the KMO and Bartlett test results for the teacher teaching quality evaluation scale. The KMO value is 0.960, which is greater than 0.7; the Bartlett test sphericity statistic is 33993.401; and the *p*-value obtained from the analysis is 0.000, which is less than the 5% significance level, indicating that factor analysis is suitable.

Table 3 presents the KMO and Bartlett's test results for the teacher teaching quality evaluation scale, and Table 4 presents the rotated component matrix. Principal component analysis was used to extract factors, and the factors were rotated using the method of maximum variance. Following the principle that the cumulative contribution rate exceeds 50% and the eigenvalue is greater than 1, 10 major factors were extracted from the scale. The cumulative variance contribution rate of these 10 major factors reached 74.986%, indicating that very little information was removed, and the factor analysis results are reliable. TQE1-TQE4 have relatively large loadings on factor 5, which can be named the learning value factor; TQE5-TQE8 have relatively large loadings on factor 3, which can be named the teacher enthusiasm factor; TQE9-TQE12 have relatively large loadings on factor 4, which can be named the organizational clarity factor; TQE13-TQE16 have relatively large loadings on factor 2, which can be named the group interaction factor; TQE17-TQE20 have relatively large loadings on factor 6, which can be named the teacher-student relationship factor; TQE2 The loadings of TQE24-1 on Factor 1 are relatively large, and this can be named the breadth of coverage factor; TQE25-TQE27 on Factor 8 are relatively large, and this can be named the exam scoring factor; TQE28-TQE29 on Factor 10 are relatively large, and this can be named the essay reading factor; TQE30-TQE32 on Factor 7 are relatively large, and this can be named the course difficulty factor; TQE33-TQE34 on Factor 9 are relatively large, and this can be named the overall evaluation factor. All factor loadings are greater than 0.5, and there is no significant cross-loading among the items. Each measurement item clusters under its corresponding factor, indicating that the scale has good construct validity.

**Table 3:** KMO and Bartlett's test for teacher teaching quality evaluation.

Kaiser–Meyer–Olkin measure of sampling adequacy		0.960
Bartlett's test of sphericity	Approx. Chi-square	33993.401
	df	561
	Sig.	0.000

**Table 4:** Rotated component matrix of the teacher's teaching quality evaluation scale.

Item	Component									
	1	2	3	4	5	6	7	8	9	10
TQE1					0.706					
TQE2					0.701					
TQE3					0.696					
TQE4					0.708					
TQE5			0.739							
TQE6			0.750							
TQE7			0.751							
TQE8			0.759							
TQE9				0.725						
TQE10				0.737						
TQE11				0.693						
TQE12				0.726						
TQE13		0.778								
TQE14		0.747								
TQE15		0.735								
TQE16		0.768								
TQE17						0.692				
TQE18						0.760				
TQE19						0.725				
TQE20						0.641				
TQE21	0.759									
TQE22	0.745									
TQE23	0.762									
TQE24	0.763									
TQE25								0.756		
TQE26								0.769		
TQE27								0.736		
TQE28										0.809
TQE29										0.804
TQE30							0.796			
TQE31							0.800			
TQE32							0.778			
TQE33									0.818	
TQE34									0.819	
Initial Eigenvalues	3.089	3.073	3.054	2.867	2.752	2.720	2.390	2.264	1.651	1.635
% of variance	9.085	9.038	8.981	8.431	8.095	8.001	7.029	6.659	4.856	4.810
Cumulative %	9.085	18.123	27.104	35.535	43.630	51.631	58.661	65.320	70.176	74.986

Table 5 shows the KMO and Bartlett test results for the school atmosphere scale. The KMO value is 0.981, which is greater than 0.7; the Bartlett test of sphericity statistic is 50977.306, and the *p*-value obtained from the analysis is 0.000, which is less than the 5% significance level, indicating that factor analysis is suitable.

**Table 5:** KMO and Bartlett's test of school atmosphere perception scale.

Kaiser–Meyer–Olkin measure of sampling adequacy		0.981
Bartlett's test of sphericity	Approx. Chi-square	50977.306
	df	703
	Sig.	0.000

Table 6 is a rotated component table of perceived school atmosphere. Five main factors can be extracted from the scale. The cumulative variance contribution rate of these five main factors reaches 71.339%, indicating that very little information was removed, and the factor analysis results are reliable. SAP1-SAP10 have large loadings on factor 1, which can be named the peer relationship factor; SAP11-SAP14 have large loadings on factor 5, which can be named the innovation factor; SAP15-SAP21 have large loadings on factor 4, which can be named the participation in decision-making factor; SAP22-SAP31 have large loadings on factor 2, which can be named the resource adequacy factor; SAP32-SAP38 have large loadings on factor 3, which can be named the student support factor. All factor loadings are greater than 0.5, and there is no serious cross-loading among the items. Each measurement item clusters under its corresponding factor, indicating that the scale has good construct validity.

**Table 6:** Rotated component matrix of the school atmosphere perception scale.

Item	Component				
	1	2	3	4	5
SAP1	0.803				
SAP2	0.791				
SAP3	0.789				
SAP4	0.790				
SAP5	0.786				
SAP6	0.791				
SAP7	0.805				
SAP8	0.790				
SAP9	0.796				
SAP10	0.809				
SAP11					0.727
SAP12					0.712
SAP13					0.724
SAP14					0.707
SAP15				0.749	
SAP16				0.700	
SAP17				0.677	
SAP18				0.688	
SAP19				0.692	
SAP20				0.673	
SAP21				0.737	
SAP22		0.796			
SAP23		0.780			
SAP24		0.786			
SAP25		0.798			
SAP26		0.785			
SAP27		0.788			
SAP28		0.807			
SAP29		0.769			
SAP30		0.789			
SAP31		0.780			
SAP32			0.792		
SAP33			0.790		
SAP34			0.802		
SAP35			0.791		
SAP36			0.779		
SAP37			0.789		
SAP38			0.803		
Initial Eigenvalues	7.380	7.372	5.313	4.449	2.594
% of variance	19.422	19.401	13.982	11.709	6.825
Cumulative %	19.422	38.823	52.805	64.514	71.339

Table 7 shows the KMO and Bartlett test results for the Academic Anxiety Scale. The KMO value is 0.980, which is greater than 0.7; the Bartlett test sphericity statistic is 39461.399, and the *p*-value obtained from the analysis is 0.000, which is less than the 5% significance level, indicating that factor analysis is suitable.

**Table 7:** KMO and Bartlett's test for academic anxiety.

Kaiser–Meyer–Olkin measure of sampling adequacy		0.980
Bartlett's test of sphericity	Approx. Chi-square	39461.399
	df	496
	Sig.	0.000

Table 8 is a rotated component scale for academic anxiety. Four main factors can be extracted from the scale. The cumulative variance contribution rate of these four main factors reaches 67.457%, indicating that very little information was removed, and the factor analysis results are reliable. AA1-AA8 have large loadings on factor 3, which can be named classroom-related emotion factors; AA9-AA16 have large loadings on factor 4, which can be named learning-related emotion factors; AA17-AA24 have large loadings on factor 1, which can be named exam-related emotion factors; and AA25-AA32 have large loadings on factor 2, which can be named essay writing-related emotion factors. All factor loadings are greater than 0.5, and there is no severe cross-loading among the items. Each measurement item clusters under its corresponding factor, indicating that the scale has good construct validity.

**Table 8:** Rotated component matrix of the academic anxiety scale.

Item	Component			
	1	2	3	4
AA1			0.769	
AA2			0.707	
AA3			0.698	
AA4			0.709	
AA5			0.699	
AA6			0.690	
AA7			0.716	
AA8			0.728	
AA9				0.747
AA10				0.638
AA11				0.615
AA12				0.662
AA13				0.652
AA14				0.679
AA15				0.676
AA16				0.713
AA17	0.789			
AA18	0.750			
AA19	0.726			
AA20	0.731			
AA21	0.761			
AA22	0.741			
AA23	0.756			
AA24	0.763			
AA25		0.738		
AA26		0.728		
AA27		0.740		
AA28		0.718		
AA29		0.729		
AA30		0.731		
AA31		0.737		
AA32		0.756		
Initial Eigenvalues	5.759	5.554	5.342	4.931
% of variance	17.998	17.358	16.692	15.409
Cumulative %	17.998	35.356	52.048	67.457

Table 9 shows the KMO and Bartlett test results for the learning motivation scale. The KMO value is 0.976, which is greater than 0.7; the Bartlett test sphericity statistic is 44516.975; and the *p*-value obtained from the analysis is 0.000, which is less than the 5% significance level, indicating that factor analysis is suitable.

**Table 9:** KMO and Bartlett's test for learning motivation.

Kaiser–Meyer–Olkin measure of sampling adequacy		0.976
Bartlett's Test of sphericity	Approx. Chi-square	44516.975
	df	435
	Sig.	0.000

Table 10 is a rotated component table of learning motivation. Six principal factors can be extracted from the scale. The cumulative variance contribution rate of these six principal factors reaches 78.432%, indicating that very little information was removed, and the factor analysis results are reliable. LM1-LM11 have large loadings on factor 1, which can be named the challenge factor; LM12-LM14 have large loadings on factor 4, which can be named the enthusiasm factor; LM15-LM21 have large loadings on factor 2, which can be named the dependence on others' evaluation factor; LM22-LM26 have large loadings on factor 3, which can be named the choice of simple tasks factor; LM27-LM28 have large loadings on factor 6, which can be named the focus on interpersonal competition factor; LM29-LM30 have large loadings on factor 5, which can be named the pursuit of reward factor. All factor loadings are greater than 0.5, and there is no serious cross-loading among the items. Each measurement item clusters under its corresponding factor, indicating that the scale has good construct validity.

**Table 10:** Rotated component matrix of learning motivation scale.

Item	Component					
	1	2	3	4	5	6
LM1	0.771					
LM2	0.766					
LM3	0.752					
LM4	0.754					
LM5	0.749					
LM6	0.764					
LM7	0.762					
LM8	0.760					
LM9	0.770					
LM10	0.770					
LM11	0.773					
LM12				0.741		
LM13				0.726		
LM14				0.759		
LM15		0.803				
LM16		0.778				
LM17		0.801				
LM18		0.786				
LM19		0.794				
LM20		0.792				
LM21		0.809				
LM22			0.808			
LM23			0.789			
LM24			0.785			
LM25			0.786			
LM26			0.804			
LM27						0.814
LM28						0.807
LM29					0.826	
LM30					0.831	
Initial Eigenvalues	8.005	5.652	4.214	2.247	1.733	1.678
% of variance	26.683	18.840	14.046	7.492	5.777	5.595
Cumulative %	26.683	45.523	59.569	67.061	72.837	78.432

### 4.2 Confirmatory factor analysis

**Figure 1:**
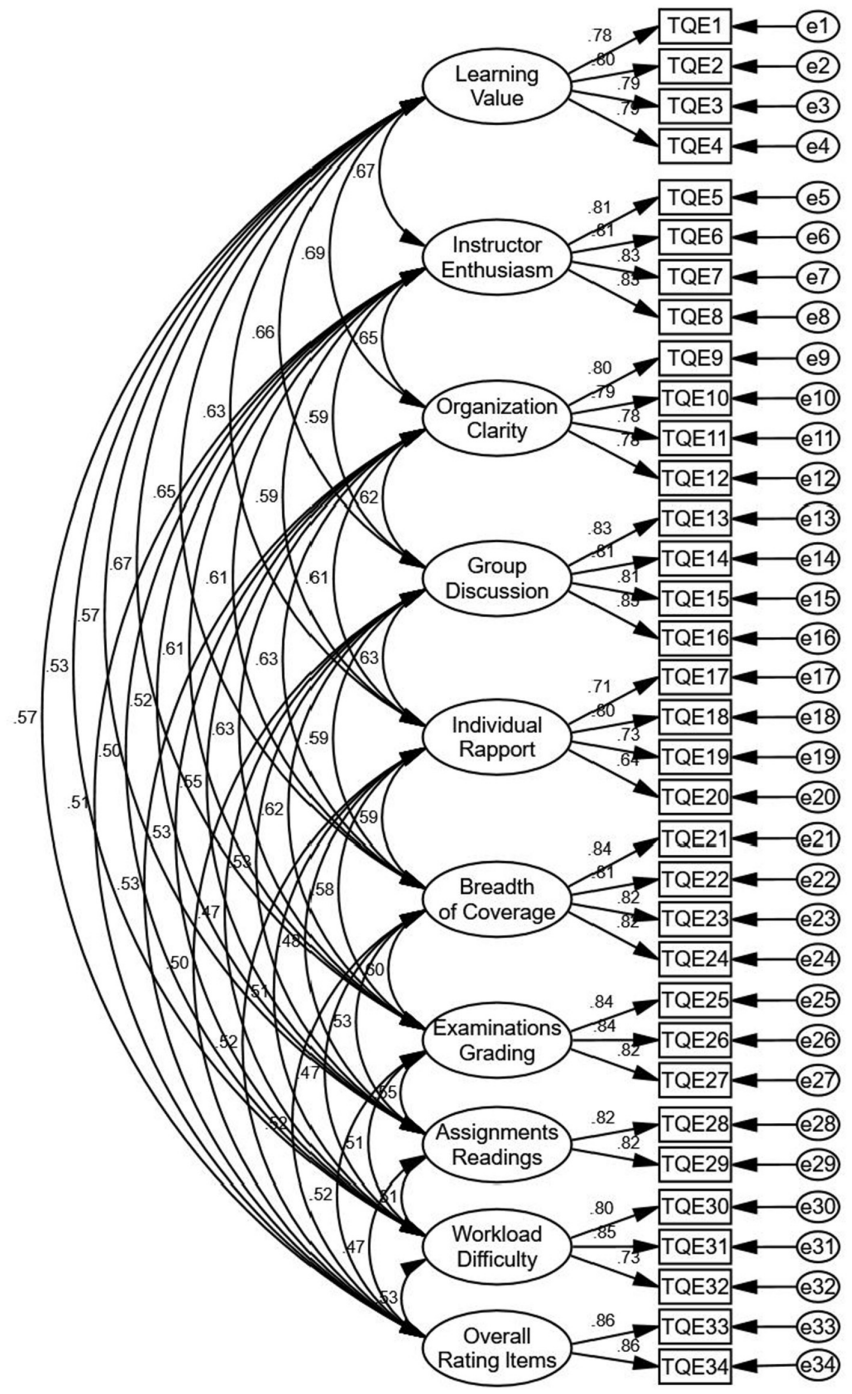
Confirmatory factor analysis of the teacher teaching quality evaluation scale.

This paper uses AMOS 24.0 software to conduct confirmatory factor analysis on the scale, primarily to verify its convergent validity and discriminant validity. Convergent validity refers to the fact that items measuring the same latent trait fall within the same factor surface, and that the measurements between items are highly correlated. It emphasizes that items that should belong to the same factor are indeed under the same factor, mainly examining the strength of the correlation among several items within the same dimension. Discriminant validity refers to the presence of low correlation or significant differences between the latent trait represented by one surface and the latent trait represented by other surfaces. It emphasizes that items that should not belong to the same factor are indeed not under the same factor, mainly examining whether there is differentiation between items across different dimensions. Convergent validity is generally determined using indicators such as factor loadings, the composite reliability scale (CR), and the mean variance extraction scale (AVE), while discriminant validity is determined by comparing the correlation coefficient of the latent variables with the square root of the AVE. Before conducting validity analysis, it is necessary to test the model's fit. There are many types of fit indices for structural equation modeling. Generally, three types of fit indices are selected: absolute fitting index, value-added fitting index, and parsimony fitting index to test the model's fit. This study selects *X*^2^/df, RMSEA, IFI, TLI, CFI, PGFI, and PNFI as evaluation indicators for model fit testing. The following are the measurement model diagram, specific indicator evaluation criteria, and fitting results (Figure 1).

Table 11 shows the model fit table for the confirmatory factor analysis of the scale. As can be seen from the table, in terms of absolute fit indices, the *X*^2^/df value is 1.337, less than 3. The RMSEA value is 0.014, less than 0.08. The absolute fit indices are ideal. In terms of value-added fit indices, the IFI value is 0.995, greater than 0.9. The TLI value is 0.994, greater than 0.9. The CFI value is 0.995, greater than 0.9. The value-added fit indices are ideal. In terms of parsimony fit indices, the PGFI value is 0.792, greater than 0.5. The PNFI value is 0.843, greater than 0.5. The parsimony fit indices are ideal. Overall, the scale indices are ideally fitted.

**Table 11:** Model fit index results of the scale.

Index	Absolute fit index		Value-added fitting index			Simplified fitting indices	
Specific categories	*X*^2/^df	RMSEA	IFI	TLI	CFI	PGFI	PNFI
Judgment criteria	< 3	< 0.08	>0.9	>0.9	>0.9	>0.5	>0.5
Fitting results	1.337	0.014	0.995	0.994	0.995	0.792	0.843

Table 12 shows the convergent validity scale. The criteria for assessing convergent validity are threefold: All standardized regression weights must be greater than 0.5; Component reliability (CR) must be greater than 0.7; Average variance extracted (Ave) must be greater than 0.5. As shown in the table, the standardized loadings of all items on the scale are greater than 0.5, meeting the criteria. The CR values for learning value, teacher enthusiasm, organizational clarity, group interaction, teacher-student relationship, breadth of coverage, exam scoring, essay reading, course difficulty, and overall evaluation are all greater than 0.7, and the Ave values are all greater than 0.5, indicating that the scale's convergent validity meets the criteria.

**Table 12:** Convergent validity of the scale.

Dimension	Questions	Standardized factor loadings	CR	AVE
Learning value	TQE1	0.776	0.868	0.623
	TQE2	0.802		
	TQE3	0.786		
	TQE4	0.792		
Teachers' enthusiasm	TQE5	0.808	0.891	0.671
	TQE6	0.809		
	TQE7	0.833		
	TQE8	0.826		
Clear organization	TQE9	0.802	0.868	0.621
	TQE10	0.793		
	TQE11	0.776		
	TQE12	0.782		
Group interaction	TQE13	0.830	0.895	0.680
	TQE14	0.810		
	TQE15	0.811		
	TQE16	0.848		
Teacher–student relationship	TQE17	0.710	0.814	0.524
	TQE18	0.802		
	TQE19	0.733		
	TQE20	0.640		
Coverage range	TQE21	0.837	0.893	0.676
	TQE22	0.808		
	TQE23	0.820		
	TQE24	0.823		
Exam scoring	TQE25	0.838	0.872	0.694
	TQE26	0.842		
	TQE27	0.819		
Essay reading	TQE28	0.820	0.804	0.672
	TQE29	0.820		
Course difficulty	TQE30	0.802	0.836	0.631
	TQE31	0.848		
	TQE32	0.728		
Overall evaluation	TQE33	0.856	0.845	0.732
	TQE34	0.855		

**Figure 2:**
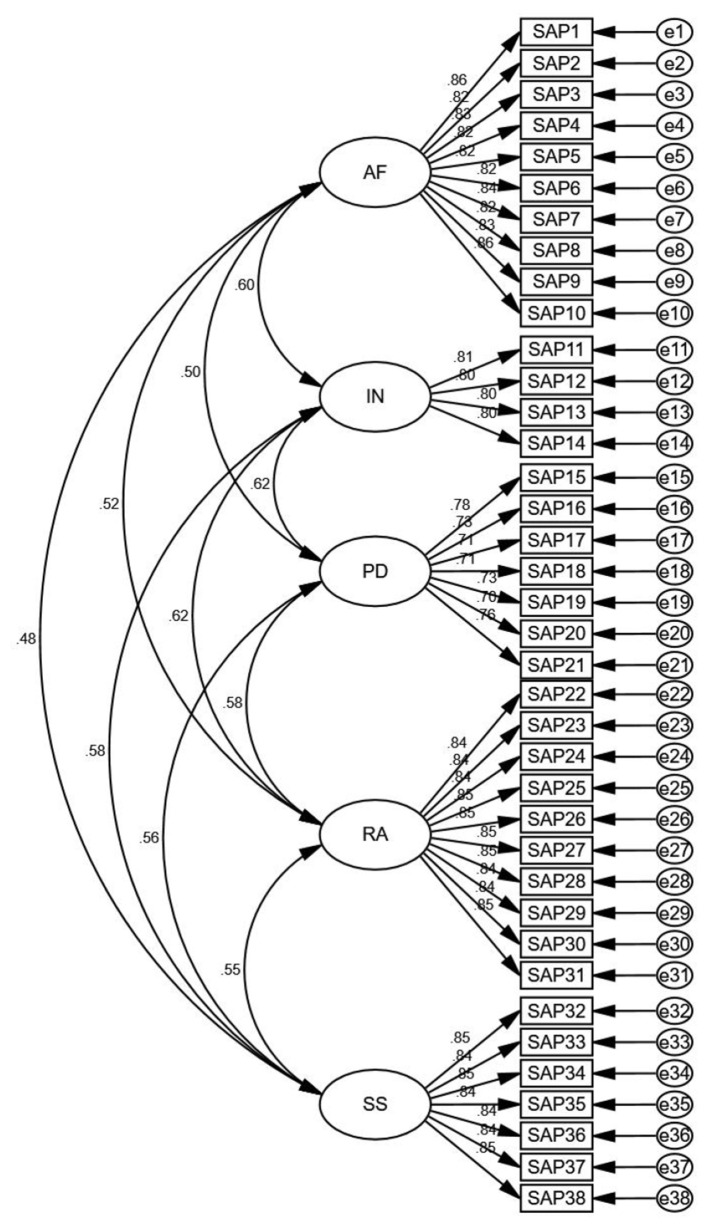
Confirmatory factor analysis of the School Atmosphere Perception Scale.

Table 13 is the discriminant validity table. The correlation coefficients between the latent variables are all less than the square root of the AVE on the corresponding diagonal, indicating that the discriminant validity between the variables is good (Figure 2).

**Table 13:** Discriminant validity scale.

Variable	1	2	3	4	5	6	7	8	9	10
1. Learning value	**0.789**									
2. Teacher's enthusiasm	0.671	**0.819**								
3. Clear organization	0.691	0.653	**0.788**							
4. Group interaction	0.657	0.592	0.62	**0.825**						
5. Teacher–student relationship	0.632	0.592	0.61	0.626	**0.724**					
6. Coverage range	0.654	0.612	0.635	0.593	0.589	**0.822**				
7. Exam scoring	0.669	0.611	0.625	0.621	0.578	0.601	**0.833**			
8. Composition reading	0.566	0.519	0.552	0.533	0.483	0.532	0.549	**0.820**		
9. Course difficulty	0.526	0.499	0.53	0.475	0.51	0.471	0.508	0.514	**0.794**	
10. Overall evaluation	0.565	0.514	0.531	0.5	0.518	0.521	0.515	0.468	0.532	**0.856**

The bold text on the diagonal represents the square root of AVE, and the text below the diagonal represents the correlation coefficients between latent variables.

Table 14 shows the model fit table for the confirmatory factor analysis of the scale. As can be seen from the table, in terms of absolute fit indices, the X^2^/df value is 1.334, less than 3. The RMSEA value is 0.014, less than 0.08. The absolute fit indices are ideal. In terms of value-added fit indices, the IFI value is 0.996, greater than 0.9. The TLI value is 0.995, greater than 0.9. The CFI value is 0.996, greater than 0.9. The value-added fit indices are ideal. In terms of parsimony fit indices, the PGFI value is 0.860, greater than 0.5. The PNFI value is 0.916, greater than 0.5. The parsimony fit indices are ideal. Overall, the scale indices are ideally fitted.

**Table 14:** Model fit index results of the scale.

Index	Absolute fit index		Value-added fitting index			Simplified fitting indices	
Specific categories	*X*^2/^df	RMSEA	IFI	TLI	CFI	PGFI	PNFI
Judgment criteria	< 3	< 0.08	>0.9	>0.9	>0.9	>0.5	>0.5
Fitting results	1.334	0.014	0.996	0.995	0.996	0.860	0.916

Table 15 shows the convergent validity of the scale. The CR values for peer relationships, innovativeness, participation in decision-making, resource adequacy, and student support are all greater than 0.7, and the AVE values are all greater than 0.5, indicating that the convergent validity of the scale meets the standard.

**Table 15:** Convergent validity of the scale.

Dimension	Questions	Standardized factor loadings	CR	AVE
Classmate relationship	SAP1	0.856	0.957	0.692
	SAP2	0.820		
	SAP3	0.831		
	SAP4	0.820		
	SAP5	0.824		
	SAP6	0.818		
	SAP7	0.838		
	SAP8	0.818		
	SAP9	0.832		
	SAP10	0.861		
Innovation	SAP11	0.813	0.881	0.648
	SAP12	0.804		
	SAP13	0.802		
	SAP14	0.802		
Participate in decision-making	SAP15	0.779	0.889	0.534
	SAP16	0.725		
	SAP17	0.706		
	SAP18	0.708		
	SAP19	0.734		
	SAP20	0.699		
	SAP21	0.759		
Resource sufficiency	SAP22	0.836	0.961	0.713
	SAP23	0.843		
	SAP24	0.843		
	SAP25	0.848		
	SAP26	0.848		
	SAP27	0.848		
	SAP28	0.852		
	SAP29	0.843		
	SAP30	0.835		
	SAP31	0.847		
Student support	SAP32	0.849	0.946	0.713
	SAP33	0.844		
	SAP34	0.848		
	SAP35	0.841		
	SAP36	0.839		
	SAP37	0.839		
	SAP38	0.850		

**Figure 3:**
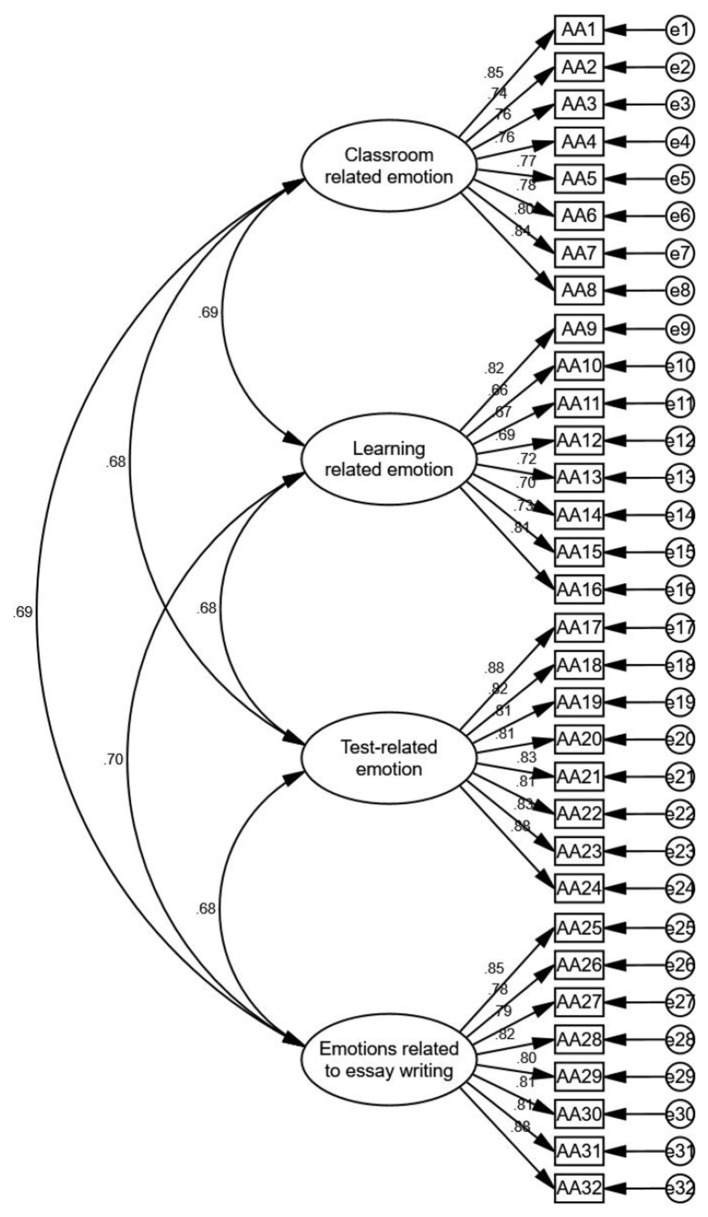
Confirmatory factor analysis of the perceived academic anxiety scale.

Table 16 is the discriminant validity table. The correlation coefficients between the latent variables are all less than the square root of the AVE on the corresponding diagonal, indicating that the discriminant validity between the variables is good (Figure 3).

**Table 16:** Discriminant validity scale.

Variable	1	2	3	4	5
1. Classmate relationships	**0.832**				
2. Innovation	0.599	**0.805**			
3. Participate in decision-making	0.499	0.617	**0.731**		
4. Resource sufficiency	0.524	0.616	0.58	**0.844**	
5. Student support	0.485	0.575	0.564	0.552	**0.844**

The bold text on the diagonal represents the square root of AVE, and the text below the diagonal represents the correlation coefficients between latent variables.

Table 17 shows the model fit table for the confirmatory factor analysis of the scale. As can be seen from the table, in terms of absolute fit indices, the *X*^2^/df value is 2.212, less than 3. The RMSEA value is 0.027, less than 0.08. The absolute fit indices are ideal. In terms of value-added fit indices, the IFI value is 0.986, greater than 0.9. The TLI value is 0.985, greater than 0.9. The CFI value is 0.986, greater than 0.9. The value-added fit indices are ideal. In terms of parsimony fit indices, the PGFI value is 0.836, greater than 0.5. The PNFI value is 0.900, greater than 0.5. The parsimony fit indices are ideal. Overall, the scale indices are ideally fitted.

**Table 17:** Model fit indicators of the scale.

Index	Absolute fit index		Value-added fitting index			Simplified fitting indices	
Specific categories	*X*^2/^df	RMSEA	IFI	TLI	CFI	PGFI	PNFI
Judgment criteria	< 3	< 0.08	>0.9	>0.9	>0.9	>0.5	>0.5
Fitting results	2.212	0.027	0.986	0.985	0.986	0.836	0.900

Table 18 shows the convergent validity of the scale. The CR values for classroom-related emotions, learning-related emotions, exam-related emotions, and paper-writing-related emotions are all greater than 0.7, and the AVE values are all greater than 0.5, indicating that the convergent validity of the scale meets the standard.

**Table 18:** Convergent validity of the scale.

Dimension	Questions	Standardized factor loadings	CR	AVE
Classroom-related emotions	AA1	0.846	0.928	0.619
	AA2	0.739		
	AA3	0.761		
	AA4	0.755		
	AA5	0.771		
	AA6	0.777		
	AA7	0.799		
	AA8	0.837		
Learning related emotions	AA9	0.824	0.899	0.528
	AA10	0.662		
	AA11	0.666		
	AA12	0.692		
	AA13	0.716		
	AA14	0.696		
	AA15	0.731		
	AA16	0.808		
Exam-related emotions	AA17	0.876	0.948	0.694
	AA18	0.822		
	AA19	0.806		
	AA20	0.806		
	AA21	0.828		
	AA22	0.814		
	AA23	0.834		
	AA24	0.877		
Emotions related to academic writing	AA25	0.848	0.942	0.670
	AA26	0.785		
	AA27	0.793		
	AA28	0.817		
	AA29	0.804		
	AA30	0.808		
	AA31	0.807		
	AA32	0.880		

**Figure 4:**
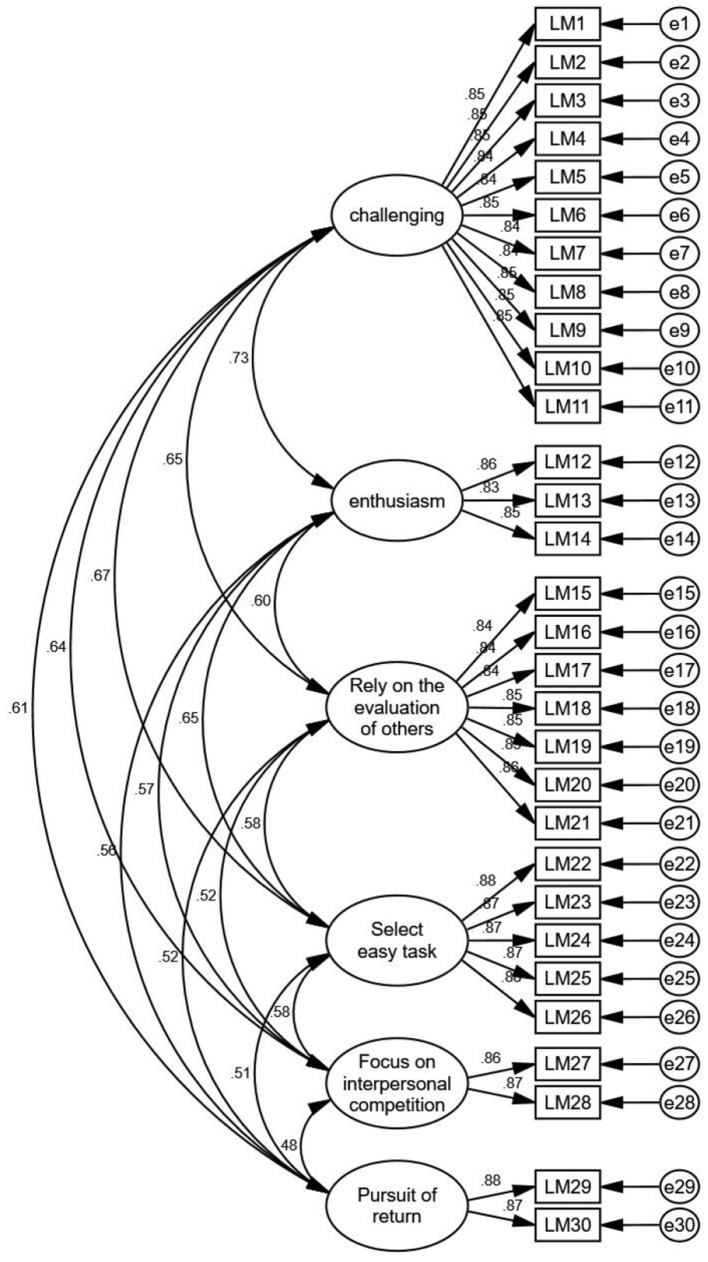
Confirmatory factor analysis of the perceived learning motivation scale.

Table 19 is the discriminant validity table. The correlation coefficients between the latent variables are all less than the square root of the AVE on the corresponding diagonal, indicating that the discriminant validity between the variables is good (Figure 4).

**Table 19:** Discriminant validity scale.

Variable	1	2	3	4
1. Classroom-related emotions	**0.787**			
2. Learning about related emotions	0.692	**0.727**		
3. Exam-related emotions	0.683	0.676	**0.833**	
4. Emotions related to thesis writing	0.691	0.696	0.683	**0.819**

The bold text on the diagonal represents the square root of AVE, and the text below the diagonal represents the correlation coefficients between latent variables.

Table 20 shows the model fit table for the confirmatory factor analysis of the scale. As can be seen from the table, in terms of absolute fit indices, the *X*^2^/df value is 1.233, less than 3. The RMSEA value is 0.012, less than 0.08. The absolute fit indices are ideal. In terms of value-added fit indices, the IFI value is 0.998, greater than 0.9. The TLI value is 0.998, greater than 0.9. The CFI value is 0.998, greater than 0.9. The value-added fit indices are ideal. In terms of parsimony fit indices, the PGFI value is 0.723, greater than 0.5. The PNFI value is 0.887, greater than 0.5. The parsimony fit indices are ideal. Overall, the scale indices are ideally fitted.

**Table 20:** Model fit index results of the scale.

Index	Absolute fit index		Value-added fitting index			Simplified fitting indices	
Specific categories	*X*^2/^df	RMSEA	IFI	TLI	CFI	PGFI	PNFI
Judgment criteria	< 3	< 0.08	>0.9	>0.9	>0.9	>0.5	>0.5
Fitting results	1.233	0.012	0.998	0.998	0.998	0.723	0.887

Table 21 shows the convergent validity of the scale. The CR values for challenge, enthusiasm, reliance on others' evaluation, choosing simple tasks, focusing on interpersonal competition, and seeking rewards are all greater than 0.7, and the AVE values are all greater than 0.5, indicating that the convergent validity of the scale meets the standard.

**Table 21:** Convergent validity of the scale.

Dimension	Questions	Standardized factor loadings	CR	AVE
Challenging	LM1	0.849	0.965	0.717
	LM2	0.846		
	LM3	0.850		
	LM4	0.840		
	LM5	0.843		
	LM6	0.854		
	LM7	0.838		
	LM8	0.844		
	LM9	0.852		
	LM10	0.848		
	LM11	0.852		
Passion	LM12	0.856	0.885	0.719
	LM13	0.834		
	LM14	0.854		
Relying on others' evaluation	LM15	0.839	0.948	0.721
	LM16	0.840		
	LM17	0.845		
	LM18	0.854		
	LM19	0.852		
	LM20	0.849		
	LM21	0.864		
Select a simple task	LM22	0.882	0.942	0.765
	LM23	0.873		
	LM24	0.867		
	LM25	0.871		
	LM26	0.881		
Focus on interpersonal competition	LM27	0.863	0.859	0.753
	LM28	0.872		
Seeking returns	LM29	0.880	0.864	0.761
	LM30	0.865		

Table 22 is the discriminant validity table. The correlation coefficients between the latent variables are all less than the square root of the AVE on the corresponding diagonal, indicating that the discriminant validity between the variables is good.

**Table 22:** Discriminant validity scale.

Variable	1	2	3	4	5	6
1. Challenging	**0.847**					
2. Enthusiasm	0.729	**0.848**				
3. Relying on the evaluation of others	0.646	0.595	**0.849**			
4. Select a simple task	0.67	0.647	0.581	**0.875**		
5. Pay attention to interpersonal competition	0.643	0.569	0.518	0.575	**0.868**	
6. Seeking returns	0.608	0.558	0.523	0.513	0.476	**0.872**

The bold text on the diagonal represents the square root of AVE, and the text below the diagonal represents the correlation coefficients between latent variables.

### 4.3 Common method bias test

Because all variables were measured using self-report questionnaires, common method bias was assessed following the recommendations of Podsakoff et al. (2003). Harman's single-factor test was conducted by entering all scale items into an unrotated exploratory factor analysis. The first unrotated factor explained 5.80% of the total variance, which is below the commonly used 40% threshold. This result suggests that common method variance was unlikely to dominate the observed associations. Nevertheless, common method bias cannot be completely excluded, so this issue is also acknowledged as a limitation.

### 4.4 Descriptive analysis

The descriptive analysis provides information such as the minimum, maximum, mean, and standard deviation of the variables, facilitating understanding of the scores of the research variables. Secondly, it provides skewness and kurtosis indices to determine whether the variables approximately follow a normal distribution. Generally, an absolute skewness value less than 3 and an absolute kurtosis value less than 10 indicate that the observed variables approximately follow a normal distribution. The results are shown in Table 23.

**Table 23:** Descriptive analysis.

Variable	*N*	Minimum	Maximum	Mean	Std. deviation	Skewness	Kurtosis
**Teacher teaching quality evaluation**	1,643	1.44	5.00	3.956	0.582	−2.032	3.982
Learning value	1,643	1.00	5.00	4.097	0.734	−1.480	1.820
Teachers' enthusiasm	1,643	1.25	5.00	4.032	0.752	−1.250	1.175
Clear organization	1,643	1.00	5.00	4.048	0.767	−1.304	1.105
Group interaction	1,643	1.00	5.00	4.006	0.836	−1.275	0.746
Teacher–student relationship	1,643	1.00	5.00	3.837	0.647	−1.186	2.015
Coverage range	1,643	1.25	5.00	4.027	0.850	−1.440	1.244
Exam scoring	1,643	1.00	5.00	4.065	0.821	−1.247	0.906
Essay reading	1,643	1.00	5.00	3.970	0.826	−1.022	0.507
Course difficulty	1,643	1.33	5.00	3.537	0.787	−0.304	−0.453
Overall evaluation	1,643	1.00	5.00	3.789	0.859	−0.615	0.038
**School atmosphere perception**	1,643	1.55	5.00	3.872	0.665	−1.303	1.344
Classmate relationship	1,643	1.40	5.00	3.506	0.944	−0.396	−0.989
Innovation	1,643	1.00	5.00	4.063	0.776	−1.194	0.698
Participate in decision-making	1,643	1.57	5.00	3.969	0.772	−1.246	0.943
Resource sufficiency	1,643	1.00	5.00	4.035	0.820	−1.342	0.627
Student support	1,643	1.43	5.00	3.958	0.853	−1.044	−0.154
**Academic anxiety**	1,643	1.00	4.66	3.446	0.842	−0.446	−1.140
Classroom-related emotions	1,643	1.00	4.88	3.436	1.005	−0.378	−1.270
Learning related emotions	1,643	1.00	5.00	3.444	0.901	−0.393	−1.037
Exam-related emotions	1,643	1.00	5.00	3.456	1.028	−0.247	−1.395
Emotions related to academic writing	1,643	1.00	5.00	3.447	1.012	−0.278	−1.328
**Learning motivation**	1,643	1.80	5.00	4.012	0.695	−1.748	2.232
Challenging	1,643	1.64	5.00	4.096	0.762	−1.574	1.560
Passion	1,643	1.00	5.00	4.026	0.909	−1.282	0.470
Relying on others' evaluation	1,643	1.14	5.00	3.966	0.852	−1.196	0.330
Select a simple task	1,643	1.00	5.00	3.953	0.939	−1.178	0.050
Focus on interpersonal competition	1,643	1.00	5.00	3.910	0.896	−0.897	0.022
Seeking returns	1,643	1.00	5.00	3.940	0.962	−0.975	0.036

Bold entries indicate the overall construct scores; non-bold entries represent the corresponding subdimensions.

Table 23 is a descriptive analysis table. The means for teacher evaluation of teaching quality, perceived school atmosphere, academic anxiety, and learning motivation are 3.956, 3.872, 3.446, and 4.012, respectively. The absolute values of the skewness of each variable and its dimensions are all less than 3, and the absolute values of the kurtosis are all less than 10, indicating that these variables approximately follow a normal distribution.

### 4.5 Correlation analysis

To verify whether there is a correlation between the variables involved in this study, Pearson correlation analysis was used. If the correlation coefficient passes the statistical significance test, it indicates that there is a significant correlation between the variables, providing a statistical basis for the subsequent regression analysis. The correlation coefficient ranges from −1 to 1. The closer the value is to 1, the stronger the positive correlation between the variables; conversely, the closer the value is to −1, the stronger the negative correlation between the variables.

Table 24 shows the correlation analysis results. Teacher evaluation of teaching quality was significantly positively correlated with school climate perception (*r* = 0.411, *P* < 0.01) and learning motivation (*r* = 0.378, *P* < 0.01), and significantly negatively correlated with academic anxiety (*r* = −0.358, *P* < 0.01). School climate perception was negatively correlated with academic anxiety (*r* = −0.266, *P* < 0.01) and positively correlated with learning motivation (*r* = 0.414, *P* < 0.01). Academic anxiety was negatively correlated with learning motivation (*r* = −0.322, *P* < 0.01). These findings provide preliminary support for the hypothesized pattern of associations. However, they do not establish causal direction; therefore, the subsequent structural equation model should also be interpreted as an association-based model.

**Table 24:** Correlation analysis.

Variable	1	2	3	4
1. Evaluation of teachers' teaching quality	1			
2. School atmosphere perception	0.411^**^	1		
3. Academic anxiety	−0.358^**^	−0.266^**^	1	
4. Learning motivation	0.378^**^	0.414^**^	−0.332^**^	1

^**^ represents *P* < 0.01.

### 4.6 Structural equation model

**Figure 5:**
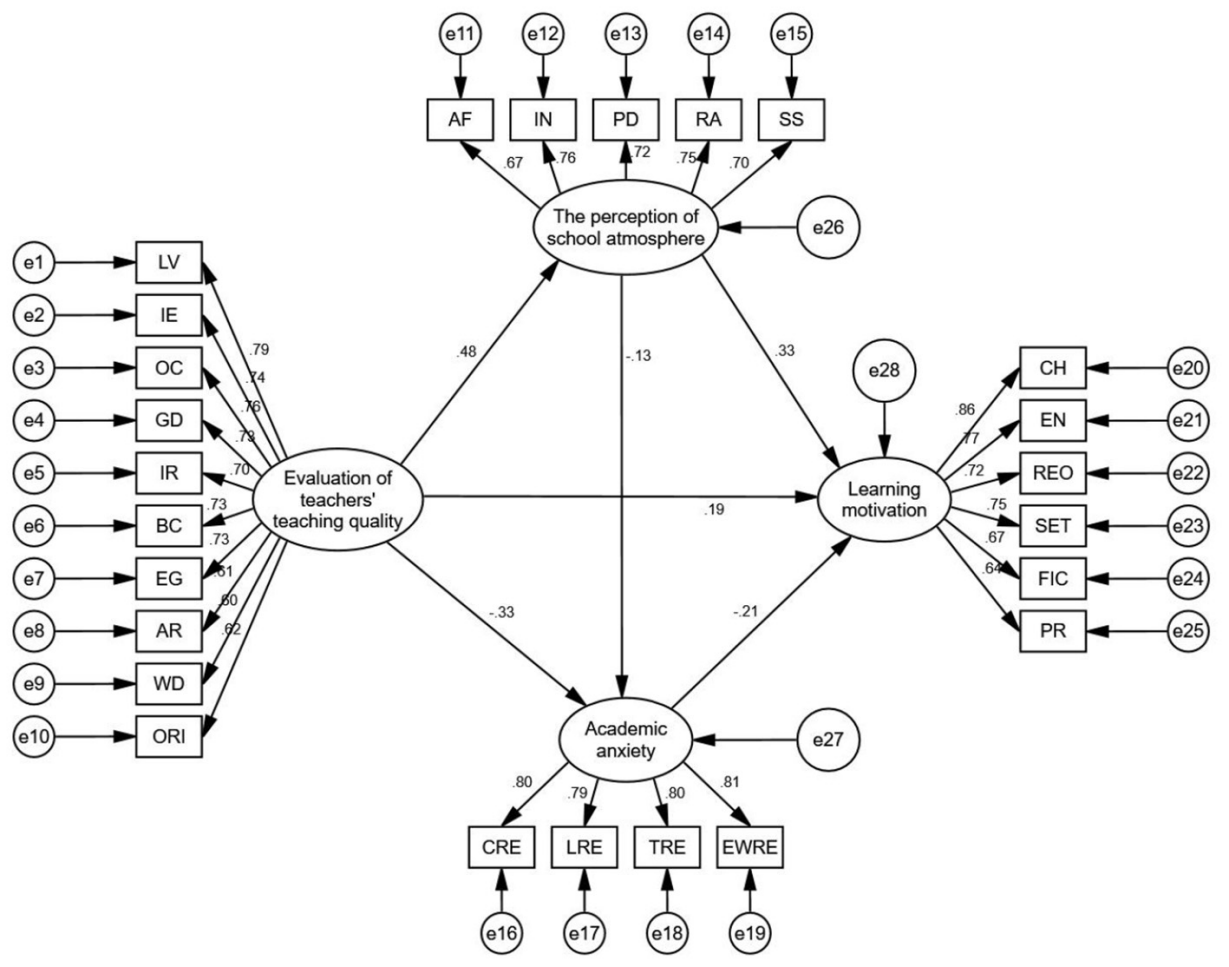
Structural equation model. The figure presents the hypothesized statistical mediation model. Teacher teaching quality evaluation is specified as the predictor, school climate perception and academic anxiety are specified as mediators, and learning motivation is specified as the outcome variable. The arrows should be interpreted as hypothesized paths within a cross-sectional SEM, not as evidence of causal direction.

The reliability analysis, exploratory factor analysis, and confirmatory factor analysis indicated that the measurement scales had acceptable reliability and validity in the present sample. AMOS 24.0 was then used to construct the structural equation model shown in Figure 5. Teacher teaching quality evaluation was specified as the predictor variable, school climate perception and academic anxiety as statistical mediators, and learning motivation as the outcome variable. Because all variables were measured at a single time point, the model estimates conditional associations and indirect pathways rather than confirmed temporal mechanisms.

Table 25 is the model fit table. As shown in the table, regarding the absolute fit indices, the *X*^2^/df value is 1.649, less than 3. The RMSEA value is 0.020, less than 0.08. The absolute fit indices are ideal. Regarding the value-added fit indices, the IFI value is 0.991, greater than 0.9. The TLI value is 0.990, greater than 0.9. The CFI value is 0.991, greater than 0.9. The value-added fit indices are ideal. Regarding the parsimony fit indices, the PGFI value is 0.810, greater than 0.5. The PNFI value is 0.876, greater than 0.5. The parsimony fit indices are ideal. Overall, the scale indices are ideally fitted.

**Table 25:** Model fit index results of the scale.

Index	Absolute fit index		Value-added fitting index			Simplified fitting indices	
Specific categories	*X*^2/^df	RMSEA	IFI	TLI	CFI	PGFI	PNFI
Judgment criteria	< 3	< 0.08	>0.9	>0.9	>0.9	>0.5	>0.5
Fitting results	1.649	0.020	0.991	0.990	0.991	0.810	0.876

Table 26 presents the path analysis results. Teacher teaching quality evaluation was positively associated with school climate perception [β = 0.480, SE = 0.033, 95% CI (0.415, 0.545), *P* < 0.001], supporting H1. Teacher teaching quality evaluation was negatively associated with academic anxiety [β = −0.333, SE = 0.044, 95% CI (−0.419, −0.247), *P* < 0.001], supporting H2. School climate perception was negatively associated with academic anxiety [β = −0.130, SE = 0.040, 95% CI (−0.208, −0.052), *P* < 0.001], supporting H3. School climate perception was positively associated with learning motivation [β = 0.330, SE = 0.032, 95% CI (0.267, 0.393), *P* < 0.001], supporting H4. Academic anxiety was negatively associated with learning motivation [β = −0.205, SE = 0.022, 95% CI (−0.248, −0.162), *P* < 0.001], supporting H5. Teacher teaching quality evaluation remained positively associated with learning motivation after the mediators were included [β = 0.185, SE = 0.034, 95% CI (0.118, 0.252), *P* < 0.001], supporting H6 and indicating partial mediation rather than full mediation.

**Table 26:** Path analysis.

Path	Estimate	SE	CR	*P*
School atmosphere perception < – Teacher teaching quality evaluation	0.480	0.033	15.868	^***^
Academic anxiety < – Teacher teaching quality evaluation	−0.333	0.044	−10.533	^***^
Academic anxiety < – School Atmosphere Perception	−0.130	0.040	−4.113	^***^
Learning motivation < – School Atmosphere Perception	0.330	0.032	10.667	^***^
Learning motivation < – Academic anxiety	−0.205	0.022	−7.495	^***^
Learning motivation < – Teacher teaching quality evaluation	0.185	0.034	6.199	^***^

^***^p < 0.001.

Bias-corrected bootstrap confidence intervals were used to assess the statistical significance of indirect effects (MacKinnon et al., 2004). Table 27 presents the bootstrap mediation results based on 5,000 repeated samples and bias-corrected 95% confidence intervals. The total association between teacher teaching quality evaluation and learning motivation was 0.424, 95% CI [0.354, 0.491], and the direct association remained significant at 0.185, 95% CI [0.113, 0.258]. The indirect association through school climate perception was 0.158, 95% CI [0.118, 0.202], supporting H7. The indirect association through academic anxiety was 0.068, 95% CI [0.050, 0.088], supporting H8. The chain pathway through school climate perception and academic anxiety was also statistically significant at 0.013, 95% CI [0.008, 0.019], supporting H9. In substantive terms, the school-climate pathway accounted for approximately 37.3% of the total association, the academic-anxiety pathway for approximately 16.0%, and the chain pathway for approximately 3.1%. Therefore, although the chain pathway is statistically reliable, its practical magnitude is small and should be interpreted cautiously. The reduction from the bivariate correlation between teaching quality evaluation and learning motivation (*r* = 0.378) to the direct SEM path (β = 0.185) suggests that part of their shared variance is accounted for by school climate perception and academic anxiety.

**Table 27:** Mediation effect test of school atmosphere perception and academic anxiety.

Effect	Estimate	SE	Lower	Upper
Total effect	0.424	0.034	0.354	0.491
direct effects	0.185	0.037	0.113	0.258
The mediating effect of school atmosphere perception	0.158	0.022	0.118	0.202
The mediating effect of academic anxiety	0.068	0.010	0.050	0.088
The chain-mediating effect of perceived school atmosphere and academic anxiety	0.013	0.003	0.008	0.019

## 5 Discussion

### 5.1 Educational implications and practical applications

This study contributes to understanding the association between pre-service English teachers' evaluation of teaching quality and their learning motivation by incorporating school climate perception and academic anxiety as mediating variables. The total association between teacher teaching quality evaluation and learning motivation was 0.424, while the indirect associations through school climate perception and academic anxiety were 0.158 and 0.068, respectively. The chain mediation effect was statistically significant but small (0.013). These results indicate partial mediation: teacher teaching quality evaluation remained directly associated with learning motivation, while school climate perception and academic anxiety explained additional portions of the association.

The findings should not be read as demonstrating that teaching quality objectively improves school climate or reduces anxiety. Rather, they show that pre-service teachers who evaluated teaching quality more positively also tended to perceive the school climate more positively, report lower academic anxiety, and report stronger learning motivation. This interpretation is consistent with the perception-based nature of the measurement instruments and the cross-sectional design. The results are also consistent with Gonţa and Tripon's (2021) argument that students' evaluations of teaching practices are shaped by their experienced match between instructional practices and learning expectations.

The strongest indirect pathway operated through school climate perception. This suggests that the wider learning context may be an important psychological and social condition associated with motivation among pre-service teachers. Academic anxiety also showed a negative association with learning motivation, indicating that students who reported greater worry and tension about academic tasks tended to report lower motivation. The chain effect was statistically significant but modest; therefore, it should be treated as a supplementary pathway rather than the central explanatory mechanism. This more cautious interpretation is important because the temporal ordering implied by chain mediation cannot be verified with same-time self-report data.

On this basis, several practical implications can be stated cautiously. Teacher education programmes may benefit from strengthening teacher-student interaction, clarifying academic expectations, and improving student support services. Schools may also consider mechanisms for monitoring students' perceptions of climate and academic-emotional pressure. These implications are not intervention effects demonstrated by the present study; rather, they are practice-oriented directions suggested by the observed associations. Future intervention or longitudinal studies are needed before firm claims can be made about whether changes in teaching quality, climate, or anxiety produce changes in learning motivation.

In the Chinese teacher-education context, students' evaluations of teaching may carry particular significance because teachers often hold strong instructional and moral authority. However, this cultural context also reinforces the need to distinguish subjective evaluation from objective teaching quality. The present findings therefore show how pre-service English teachers' perceptions cluster together; they do not independently verify teachers' actual classroom performance.

### 5.2 Feedback literacy as a future research direction

Feedback literacy has also attracted increasing attention in foreign-language education research (Dong and Gao, 2022). The concept of feedback literacy is relevant to teacher education, but it was not directly measured in the present study. Feedback literacy generally refers to learners' capacity to understand, evaluate, use, and reflect on feedback in ways that support learning (Sutton, 2012; Carless and Boud, 2018). For pre-service teachers, this concept may be especially important because they must learn both how to use feedback as learners and how to provide feedback as future teachers.

Accordingly, this study no longer presents pre-service teacher feedback literacy as an empirical finding. Instead, it is positioned as a future research direction suggested by the observed relationship between teaching quality evaluation and learning motivation. Future studies could measure feedback literacy directly and test whether it moderates or mediates the association between teaching evaluation experiences and professional learning outcomes.

Future research may also use qualitative interviews or mixed-method designs to examine how pre-service teachers interpret feedback from instructors, how they transform evaluation experiences into professional learning, and how feedback literacy develops during practicum or thesis-writing stages.

### 5.3 Methodological limitations and future research directions

This study has several limitations. First, the cross-sectional design prevents any firm conclusion about causal or temporal ordering. Although the SEM and bootstrap results are consistent with the proposed mediation model, alternative directions remain plausible. For example, highly motivated students may evaluate teaching quality more positively, and anxious students may perceive the school climate more negatively. Second, all variables were measured through self-report questionnaires at one time point, creating the possibility of common method bias. Harman's single-factor test did not indicate severe common method variance, but this test cannot fully rule out method effects. Third, the SLEQ and AEQ were adapted for the present context. Although reliability, EFA, and CFA results supported the revised structures, further validation is needed before the adapted scales are used in other populations. Fourth, demographic variables were collected to describe the sample and assess its composition, but they were not incorporated as control variables because the primary objective was to test the theoretical mediation model. Future research could examine gender, grade level, institutional type, internship experience, and family background as controls or moderators. Fifth, the sample consisted of Chinese pre-service English teachers, which limits generalizability to other teacher-education contexts, academic disciplines, and cultural settings.

## 6 Conclusion

This paper examined the associations among pre-service English teachers' evaluations of teaching quality, school climate perception, academic anxiety, and learning motivation. The results supported a partial mediation model in which school climate perception and academic anxiety were statistically significant mediators, and the chain pathway was significant but small. The findings suggest that pre-service teachers who report more positive teaching quality evaluations also tend to report a more supportive school climate, lower academic anxiety, and stronger learning motivation.

The study also clarifies that feedback literacy should be treated as a future research direction rather than an empirically tested construct in the present model. Subsequent studies could directly measure pre-service teacher feedback literacy and examine how it interacts with teaching quality evaluation, academic emotion, and motivation over time.

Overall, the study provides association-based evidence from a large Chinese pre-service English teacher sample. Its contribution lies in showing how teaching quality evaluation, perceived climate, academic anxiety, and motivation are statistically connected, while also identifying the methodological caution needed when interpreting cross-sectional mediation results.

## Data Availability

The raw data supporting the conclusions of this article are not publicly available because the dataset contains individual-level questionnaire responses from human participants, and consent for public data sharing was not obtained. De-identified data may be made available by the corresponding author upon reasonable request, subject to ethical and privacy considerations.
